# The Molecular Mechanism Underlying the Therapeutic Effect of Dihydromyricetin on Type 2 Diabetes Mellitus Based on Network Pharmacology, Molecular Docking, and Transcriptomics

**DOI:** 10.3390/foods13020344

**Published:** 2024-01-22

**Authors:** Xinnian Wen, Chenghao Lv, Runze Zhou, Yixue Wang, Xixin Zhou, Si Qin

**Affiliations:** 1 Laboratory of Food Function and Nutrigenomics, College of Food Science and Technology, Hunan Agricultural University, Changsha 410128, China; wenxinnianzzp@163.com (X.W.); zrz1037115096@outlook.com (R.Z.); wangyxue2000@163.com (Y.W.); 2 College of Bioscience and Biotechnology, Hunan Agricultural University, Changsha 410128, China; lvchenghao0514@163.com

**Keywords:** dihydromyricetin, T2DM, network pharmacology, molecular docking, transcriptomics

## Abstract

Type 2 diabetes mellitus (T2DM) is a chronic and complex disease, and traditional drugs have many side effects. The active compound dihydromyricetin (DHM), derived from natural plants, has been shown in our previous study to possess the potential for reducing blood glucose levels; however, its precise molecular mechanism remains unclear. In the present study, network pharmacology and transcriptomics were performed to screen the molecular targets and signaling pathways of DHM disturbed associated with T2DM, and the results were partially verified by molecular docking, RT-PCR, and Western blotting at in vivo levels. Firstly, the effect of DHM on blood glucose, lipid profile, and liver oxidative stress in db/db mice was explored and the results showed that DHM could reduce blood glucose and improve oxidative stress in the liver. Secondly, GO analysis based on network pharmacology and transcriptomics results showed that DHM mainly played a significant role in anti-inflammatory, antioxidant, and fatty acid metabolism in biological processes, on lipoprotein and respiratory chain on cell components, and on redox-related enzyme activity, iron ion binding, and glutathione transferase on molecular functional processes. KEGG system analysis results showed that the PI3K-Akt signaling pathway, IL17 signaling pathway, HIF signaling pathway, MAPK signaling pathway, AGE-RAGE signaling pathway in diabetic complications, and TNF signaling pathway were typical signaling pathways disturbed by DHM in T2DM. Thirdly, molecular docking results showed that VEGFA, SRC, HIF1A, ESR1, KDR, MMP9, PPARG, and MAPK14 are key target genes, five genes of which were verified by RT-PCR in a dose-dependent manner. Finally, Western blotting results revealed that DHM effectively upregulated the expression of AKT protein and downregulated the expression of MEK protein in the liver of db/db mice. Therefore, our study found that DHM played a therapeutic effect partially by activation of the PI3K/AKT/MAPK signaling pathway. This study establishes the foundation for DHM as a novel therapeutic agent for T2DM. Additionally, it presents a fresh approach to utilizing natural plant extracts for chemoprevention and treatment of T2DM.

## 1. Introduction

Diabetes mellitus (DM) is a chronic and complex multi-system disease. The IDF Diabetes Atlas shows that the global diabetes prevalence in 2021 was estimated to be 10.5% (536.6 million people), rising to 12.2% (783.2 million) in 2045, indicating that DM is likely to become the largest systemic metabolic disease in the world [1]. The most common is type 2 diabetes (T2DM), which accounts for up to 90% [2]. Insulin resistance (IR) is the pathological basis and core link of the pathogenesis of T2DM, which exists in the whole process of the occurrence and development of T2DM [3]. In recent years, through in vivo and in vitro experimental studies, it has been confirmed that the PI3K/Akt signaling pathway, MAPK signaling pathway, PPAR signaling pathway, oxidative stress, and inflammation-related signaling pathway are the key pathways to regulate IR, which are closely related to the pathogenesis of T2DM [4,5,6,7]. At present, patients still need to take anti-diabetic drugs to control blood glucose levels. However, side effects such as hypoglycemia, heart failure, and osteoporosis also occur, limiting its clinical application [8]. Natural products (NPs), including herbal formulations and their extracts, have been increasingly used for the treatment of T2DM. Numerous studies have shown that herbs and their active ingredients have hypoglycemic effects with low toxicity and few adverse reactions [9].

Dihydromyricetin (DHM) is the main active component of vine tea (*Ampelopsis grossedentata* (Hand.-Mazz.) W. T. Wang). DHM possesses a remarkable abundance of phenolic hydroxyl groups, rendering it highly susceptible to various chemical reactions including oxidation, dehydrogenation, esterification, and complexation with transition metal ions. DHM exhibits a diverse range of biological activities, including anti-inflammatory and anti-tumor effects, regulation of blood lipid levels, reduction of blood glucose levels, and hepatoprotective properties [10]. The bioavailability of DHM has also been greatly improved in recent years. A study has found that DHM (1.0 g and 0.5 g/kg, BW) could reduce fasting blood glucose, serum insulin, glycosylated hemoglobin levels, and HOMA-IR index, and upregulate IRS-1 (Y612) tyrosine phosphorylation, finally improving insulin resistance in fatty *db^−^/db^−^* mice [11]. DHM also could significantly increase the protein expressions of p-Akt, p-IRS-1, and p-AMPK, and improve insulin sensitivity in the liver [12]. Furthermore, there are studies that demonstrate that DHM targeted 14 potential genes in NAFLD, and PPARG and CASP3 were two hub genes for DHM against Non-alcohol fatty liver disease (NAFLD) in high-fat diet (HFD) rats [13]. In addition, studies have shown that DHM could treat diabetes and glucose metabolism disorders mainly by regulating redox and inflammation-related signaling pathways, upregulating the ratio of *Firmicus*/*Bacteroides* (F/B), and increasing the relative abundance of beneficial bacteria degree [14]. The administration of DHM has been demonstrated to have a hypoglycemic effect and improve insulin resistance, exhibiting promising potential in the management of diabetes and glucose metabolism disorders. However, in-depth and systematic molecular mechanism studies are still lacking. 

In this study, network pharmacology and transcriptomics were applied to systematically explore the molecular targets, biological processes, and signaling pathways disturbed by DHM in *db*/*db* mice at the molecular level, and the target proteins of related signaling pathways were verified by molecular docking, RT-PCR, and Western blotting. This study provides a new strategy for the chemoprevention and treatment of T2DM.

## 2. Materials and Methods

### 2.1. Materials

DHM (98%) was provided by Hunan Tea Industry Group Co. Ltd. (Changsha, China). The specific extraction process is as follows: a certain amount of rattan tea raw materials are crushed; the mixture is then extracted with hot water at 95 °C for 60 min, using a solid–liquid ratio of 1:15. The resulting solution is filtered and the filtrate is collected for further use, while the filter residue undergoes another extraction with 95 °C water to ensure complete extraction. The second filtrate is combined with the first one and finally filtered using a filter bottle. The extract was concentrated, cooled, crystallized, and subsequently filtered. The crude extract was obtained by vacuum drying. The crude extract was dissolved in hot water and then filtered while still hot. The resulting filtrate was cooled and left to crystallize in a refrigerator at temperatures ranging from 0 °C to 4 °C for over 24 h. These procedures were repeated for a total of 4 to 5 times before the final filtration step. Finally, the obtained product underwent vacuum drying to yield purified DHM with a purity of 98% (Figure S1).

### 2.2. Network Pharmacology Analysis

#### 2.2.1. Prediction of DHM Targets

The chemical structure of DHM was obtained on the PubChem platform (https://pubchem.ncbi.nlm.nih.gov/, accessed on 10 May 2023), and using the CAS number of DHM. The target of MY was obtained in the Swiss Target Prediction database (http://www.swissadme.ch/ accessed on 14 May 2023), and using the chemical structure of DHM. The chemical formula of DHM was obtained in the Swiss Target Prediction database (http://www.swissadme.ch/, accessed on 14 May 2023). Then the target validations were performed with the Uniprot database (https://www.uniprot.org/, accessed on 15 May 2023).

#### 2.2.2. Prediction of Targets of T2DM

Using “type 2 diabetes”, “T2DM”, and “diabetes mellitus type 2” as keywords, we searched for disease-related genes in the database GeneCards (http://www.genecards.org/, accessed on 18 May 2023), OMIM (http://www.omim.org/, accessed on 18 May 2023), and Therapeutic Target Database (https://db.idrblab.net/ttd/, accessed on 18 May 2023), then sorted out and screened the retrieved disease targets and delete duplicate targets.

#### 2.2.3. PPI Network Construction and Its Visualization

Using Venny 2.1 (https://bioinfogp.cnb.csic.es/tools/venny/index.html, accessed on 21 May 2023), and we drew the Wayne diagram of the component target and the disease target. We then imported the shared gene target into the STRING database (https://cn.string-db.org/, accessed on 25 May 2023) with species set to “Homo sapiens”, “minimum required interaction score” set to “medium confidence (0.4)”, and hid isolated proteins in the network. The protein–protein interaction core network (PPICN) can be obtained, and the obtained protein interaction information can be imported into the Cytoscape 3.9.1 software. The PPI network can be visually analyzed, and the “component-target-disease” network can be built using the Cytoscape 3.9.1 software.

#### 2.2.4. GO Enrichment and KEGG Pathway Analysis

The common targets of DHM and T2DM were uploaded to the DAVID 6.8 platform (https://david.ncifcrf.gov, accessed on 31 May 2023). GO function annotation and KEGG enrichment analysis (*p* < 0.01) were performed. The species type was limited to “Homo sapiens”, and the enrichment results were visually analyzed.

#### 2.2.5. Molecular Docking

The active ingredient dihydromyricetin was used as a ligand, ranked according to the magnitude of the degree value, combined with relevant literature reports, and 6 targets were selected as receptors for molecular docking. The RCSB PDB database (https://www.rcsb.org/, accessed on 15 June 2023) and was used to download the molecular structure of the receptor, and the species was selected as a human, with high credibility molecules. The active ingredient (small molecule ligand) stereostructure was downloaded from the PubChem database (https://pubchem.ncbi.nlm.nih.gov/, accessed on 15 June 2023). First, we used Pymol 2.3.0 software to pretreat them and then used Autodock Vina 1.2.2 software for molecular docking, dehydrating, hydrogenation, charge, and other operations on acceptors and ligands, and calculated the binding energy according to the hydrogenation reaction of proteins and small molecule ligands. It is generally believed that the system docking result is less than 0 to indicate that there is a certain binding activity between the ligand and the acceptor molecule, and when the ligand and the acceptor bind more closely, the greater the energy released, the smaller the binding energy. The reliability of the network analysis was evaluated and the results were visualized by PyMol software. 

### 2.3. Animals and Models

A total of 40 male SPF db/db mice and 10 male SPF C57BL/6 mice were obtained from Hunan Jingda Experimental Animal Co., Ltd. (Changsha, China) (SCXK (Xiang) 2019-0004) and raised in the barrier environment of Hunan Drug Safety Evaluation Research Center (SYXK (Xiang) 2015-0016) (Ethics Committee Name: Hunan Provincial Research Center for Safety Evaluation of Drugs. Approval Code: IACUC-2020(3)072). The animals were randomly assigned to the Control group (Control), Metformin group (MET), Low-dose DHM group (150 mg/kg, LDHM), and High-dose DHM group (300 mg/kg, HDHM), with 10 animals in each group. Additionally, a Normal group consisting of 10 C57BL/6 mice of the same age was selected, with blood glucose levels ranging from 3.1 to 7.2 mmol/L and body weights between 19.3 and 23.1 g. Each dose group received the corresponding drugs via oral gavage at a dosage of 20 mL/kg, once daily for a duration of 11 weeks. The Normal group and the Control group were administered an equivalent volume of the vehicle via oral gavage. The blood glucose levels were monitored on a weekly basis throughout the duration of the experiment, blood samples were collected from the tail tip after a fasting period of 12 h to measure fasting blood glucose levels using blood glucose detection strips and Blood Glucose Meter (Changsha Sinocare Inc., Changsha, China). Every two weeks, blood samples were collected from the posterior orbital venous plexus of the mice in each group, and centrifuged at 3000 rpm for 10 min to obtain serum. Serum levels of LDL-C, TC, TG, and HDL-C were measured using Assay Kits (FUJIFILM Wako Pure Chemical Corporation, Tokyo, Japan). The mice were administered chloral hydrate for anesthesia induction, followed by blood collection for euthanasia purposes. The liver tissues (0.1 g) were homogenized in an ice bath, followed by extraction with 1 ml of the corresponding extractant on ice for 1 h. Subsequently, centrifugation was performed at 8000 rpm for 10 min at 4 °C. The resulting supernatant was collected to determine the levels of hepatic reactive oxygen species (ROS), glutathione peroxidase (GSH-Px), aspartate aminotransferase (AST), and alanine aminotransferase (ALT) by using Assay Kits (Beijing Solarbio Science & Technology Co., Ltd., Beijing, China) according to the provided instructions. 

### 2.4. Transcriptome Sequencing Analysis

NanoDrop ND-1000 was used for quality control of the amount and purity of total RNA, and the integrity of RNA was detected by a Bioanalyzer 2100. At the same time, RNA concentration was quantified by the agarose electrophoresis program. The total RNA was >1 μg. Oligo (dT) beads were used to purify the mRNA with polyadenylate (PolyA) and specifically capture it. The captured mRNA was fragmented by a magnesium ion fragmentation kit at 94 °C for 5–7 min at high temperatures. The obtained fragmented RNA was reverse transcribed into cDNA by reverse transcriptase. *E. coli* DNA polymerase I and RNase H were used to convert these compound double-stranded DNA and RNA into DNA double-stranded DNA. At the same time, dUTP Solution was added to the double-stranded DNA, and the ends of the double-stranded DNA were complemented to flat ends and an A base was added to each end so that it could be attached to a linker with a T base at the end. Then, the oligo (dT) beads were used to screen and purify the fragments. Predenaturation was maintained at 95 °C for 3 min, followed by 8 cycles of 15 s each at 98 °C, annealing to 60 °C for 15 s, extension at 72 °C for 30 s, and final extension at 72 °C for 5 min to form a fragment size of 300 bp ± 50 bp library. Finally, two-end sequencing was performed using an Illumina NovaSeq™ 6000 (Illumina, Inc., Hayward, CA, USA) following standard procedures in PE150 mode.

### 2.5. qRT-PCR

Total RNA was extracted and purified from the liver according to the instructions of the RNA extraction kit (Hangzhou METgen Biotechnology Co., Ltd., Hangzhou, China). Then, the quantity and purity of total RNA were controlled by NanoDrop ND-1000 (Thermo Fisher Scientific Inc. Waltham, MA, USA) to ensure an RNA concentration of OD260/280>1.8. The total RNA extract was obtained. The primers were designed according to the target genes to be verified and sent to Sangon Bioengineering Co., Ltd. (Shanghai, China) for primer synthesis. Primer sequences are listed in Table S1. Total RNA was reverse transcribed to cDNA using a reverse transcription kit and stored at −20 °C. The reverse transcription reaction program was as follows: predenaturation at 37 °C for 15 min; denaturation at 85 °C for 5 s; cycle at 4 °C. cDNA was amplified by two-step PCR using CFX96 Real-Time PCR Detection System (BIO-RAD, Hercules, CA, USA). The reaction procedure was as follows: predenaturation at 95 °C for 1 min; there were 39 cycles of denaturation at 95 °C for 20 s and annealing at 60 °C for 1 min. The resulting data were exported and analyzed.

### 2.6. Western Blotting

The liver was homogenized using a modified RIPA buffer and proteinase inhibitor cocktail (Beijing Solarbio Science & Technology Co. Ltd., Beijing, China) in a homogenizer (Tiangen Biotech Co. Ltd., Beijing, China). The protein concentration was determined using the BCA Protein Concentration Determination Kit (Beijing Solarbio Science & Technology Co. Ltd., Beijing, China). Protein extracts were separated by 10% SDS-PAGE and transferred to PVDF membranes (Amershan Pharmacia Biotech, Little Chalfont, UK). The membranes were blocked at room temperature for 1 h with 5% non-fat dry milk and then incubated overnight at 4 °C with each primary antibody. Primary antibodies against AKT and MEK were obtained from Sigma-Aldrich and used at a dilution of 1:1000; antibodies against GAPDH were obtained from Sigma-Aldrich and were used at a dilution of 1:5000. Afterward, they were further incubated for 1 h with an HRP-conjugated secondary antibody. The secondary antibodies were anti-rabbit (Sigma-Aldrich, St. Louis, MO, USA) and were used at a dilution of 1:8000. Bound antibodies were detected using the ECL system with a Lumi Vision PRO machine (Buckinghamshire, UK).

### 2.7. Statistical Analysis

StringTie 2.2.0 software was used to perform FPKM quantification of gene or transcript library data, and the R package edgeR was used to analyze the differentially expressed genes between samples, with differences >2 times or <0.5 times (*p* < 0.05). DAVID (https://david.ncifcrf.gov/, accessed on 31 May 2023) was used to perform GO and KEGG enrichment analysis of genes.

Each independent experiment and data were analyzed and processed by SPSS26.0 software. Finally, GraphPad Prism 8 was used for graphics and text. All results are expressed as the mean of the number of experiments ± standard error of the mean (SEM). Significance analysis was performed using one-way ANOVA with multiple comparisons, and a statistical probability of *p* < 0.05 was considered statistically significant (* *p* < 0.05, ** *p* < 0.01, *** *p* < 0.001,**** *p* < 0.0001).

## 3. Results

### 3.1. Prediction of DHM and T2DM Targets

**Figure 1:**
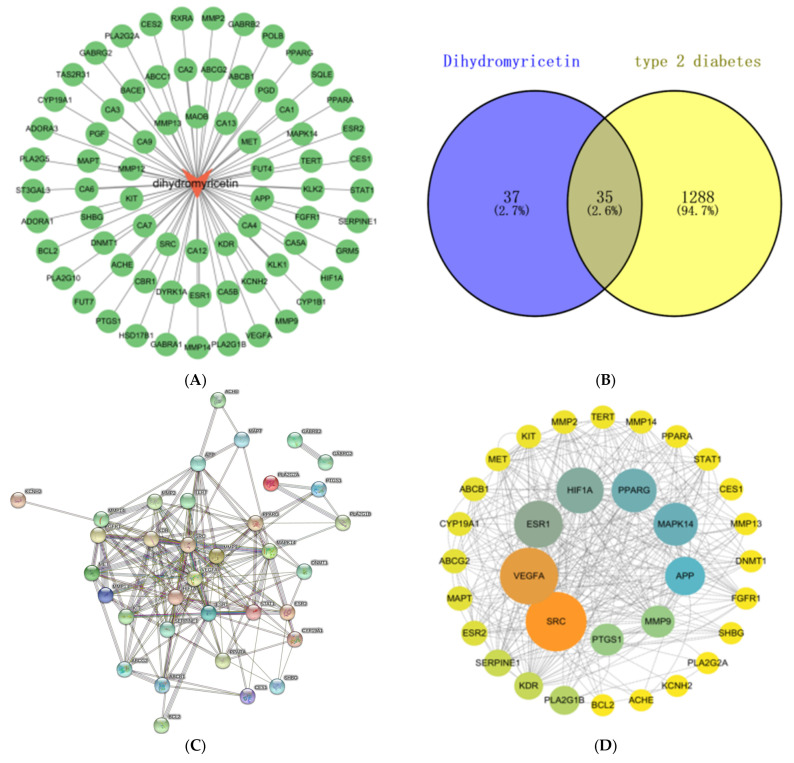

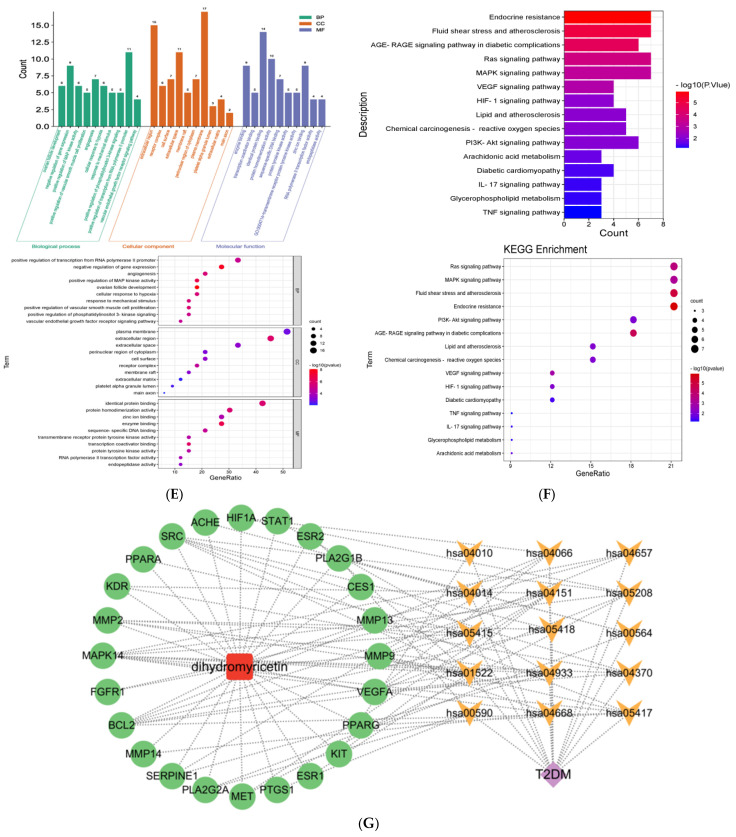
Network pharmacology analysis of DHM treatment in T2DM. (**A**) Component-target map. (**B**) Venn diagram of targets of DHM and T2DM. (**C**) PPI network diagram of DHM-related T2DM. (**D**) Information network of protein interaction. (**E**) GO enrichment analysis. (**F**) KEGG pathway enrichment analysis. (**G**) Network diagram of “composition-target-T2DM-pathway” of DHM.

We utilized the Swiss Target Prediction database to predict DHM targets, resulting in the identification of 100 potential targets. The threshold was set as probability > 0, resulting in the identification of 72 targets after screening. Subsequently, these targets were imported into Cytoscape 3.9.1 software to construct the component-target map, as depicted in Figure 1A. The GeneCards database yielded a total of 15,669 potential targets associated with T2DM, wherein a restriction of relevance score > 30 was applied, and 1116 candidate genes were ultimately identified. The 296 potential targets were retrieved from the OMIM database and the 88 potential targets were obtained from the Therapeutic Target Database. After eliminating duplicate targets, a total of 1323 potential targets associated with T2DM were acquired.

### 3.2. PPI Network Construction and Its Visualization

As shown in Figure 1B, a total of 72 targets of DHM and 1323 targets of T2DM were plotted into a Venn diagram, and 35 common targets were obtained. The above 35 common targets were imported into the STRING database to obtain the interaction relationship between proteins and proteins, and the protein interaction information network diagram was obtained. As shown in Figure 1C, the PPI network contains 35 nodes and 168 edges.

The network diagram of the interaction information of the common gene target protein was imported into the Cytoscape 3.9.1 software for network topology analysis. As shown in Figure 1D, after removing the dispersion points with low correlation, the PPI network was constructed according to the betweenness value and the degree value, which contains 33 nodes with 163 edges. The size of the node in the graph is adjusted according to the size of the degree value, and the gradient of the color is adjusted by betweenness. The larger and darker the node, the greater the degree of association of the protein with other proteins in the entire network, and the greater the likelihood that the target will function. The mediation centrality, closeness, and degree of the node are shown in Table S2.

### 3.3. GO Enrichment and KEGG Pathway Analysis Based on Network Pharmacology

The GO functional classification and enrichment analysis, as depicted in Figure 1E, identified a total of 625 significantly enriched terms (*p* < 0.05). These included 205 terms associated with biological processes (BP), 27 terms related to cellular components (CC), and 57 terms pertaining to molecular functions (MF). In BP, the 10 most significant items were ovarian follicle development, negative regulation of gene expression, positive regulation of MAP kinase activity, positive regulation of vascular smooth muscle cell proliferation, angiogenesis, cellular response to hypoxia, response to mechanical stimulus, positive regulation of phosphatidylinositol 3-kinase signaling, positive regulation of transcription from RNA polymerase II promoter, and vascular endothelial growth factor receptor signaling pathway. In CC, the 10 most significant items were extracellular region, receptor complex, cell surface, extracellular space, membrane raft, perinuclear region of cytoplasm, plasma membrane, platelet alpha granule lumen, extracellular matrix, and main axon. In MF, the 10 most significant items were enzyme binding, transcription coactivator binding, identical protein binding, protein homodimerization activity, sequence-specific DNA binding, protein tyrosine kinase activity, transmembrane receptor protein tyrosine kinase activity, zinc ion binding, RNA polymerase II transcription factor activity, and endopeptidase activity.

The KEGG pathways were significantly enriched as shown in Figure 1F; a total of 48 pathways were screened out (*p* < 0.01). The signaling pathways associated with T2DM mainly include the PI3K-Akt signaling pathway, IL17 signaling pathway, HIF-1 signaling pathway, MAPK signaling pathway, endocrine resistance, AGE-RAGE signaling pathway in diabetic complications, TNF signaling pathway, glycerophospholipid metabolism, diabetic cardiomyopathy, arachidonic acid metabolism, etc. The genes associated with these signaling pathways are shown in Table 1.

### 3.4. Construction and Analysis of “Composition-Target-Disease-Pathway” Network

As shown in Figure 1G, the targets are highlighted in green, the chemical composition is indicated in red, the disease is represented in purple, and the top 15 significant pathways are depicted in yellow. The primary protein targets of DHM in the treatment of T2DM encompass MAPK14, BLC2, SRC, MET, PPARG, VEGFA, and MMP9. Meanwhile, the MAPK signaling pathway (hsa04010), AGE-RAGE signaling pathway in diabetic complications (hsa04933), PI3K-Akt signaling pathway (hsa04151), Ras signaling pathway (hsa04014), and endocrine resistance (hsa01522) are the main signaling pathways of DHM intervention in T2DM.

### 3.5. Molecular Docking

**Figure 2:**
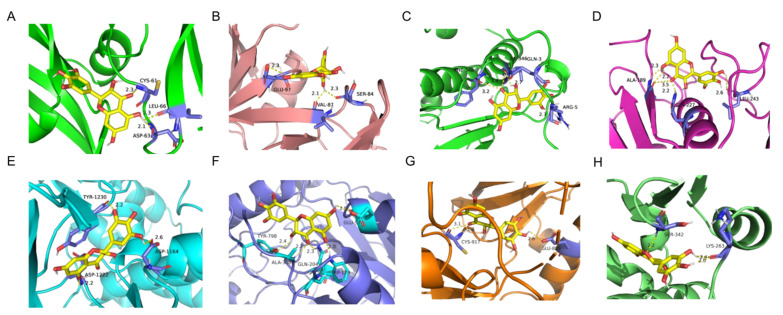
Molecular docking results of DHM and key targets. (**A**) DYM-VEGFA. (**B**) DYM-SRC. (**C**) DYM-MAPK14. (**D**) DYM-MMP9. (**E**) DYM-MET. (**F**) DYM-HIF1A. (**G**) DYM-KDR. (**H**) DYM-PPARG.

As shown in Table 2 and Figure 2, DHM has significant excellent binding activity with key targets such as VEGFA, SRC, MMP9, MAPK14, MET, HIF1A, KDR, and PPARG. Among them, the affinity of MMP9 with DHM was the highest, which was −10.2 kcal/mol; the binding sites were ALA-189, LEU-243, and GLN-227. The affinity of MAPK14 was −9.0 kcal/mol; the binding sites were PHE-348, ARG-5, GLN-3, and ILE-346. The affinity of PPARG was −8.2 kcal/mol; the binding sites were LYS-263 and SER-342. The affinity of MET was −7.9 kcal/mol; the binding sites were TYR-1230, ASP-1164, and ASP-1222. The affinity of HIF1A was −7.5 kcal/mol; the binding sites were TYR-798, GLU-57, ALA-300, GLN-204, and TRP-179. The affinity of KDR was −7.5 kcal/mol; the binding sites were CYS-917 and GLU-883. The affinity of VEGFA was −7.0 kcal/mol; the binding sites were CYS-61, LEU-66, and ASP-63. The affinity of SRC was −6.4 kcal/mol; the binding sites were GLU-97, SER-84, and VAL-87.

### 3.6. Effects of DHM on Physiological and Biochemical Indicators of db/db Mice

**Figure 3:**
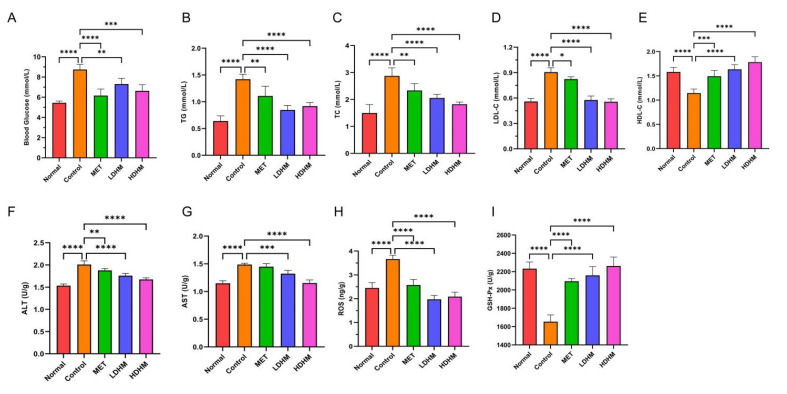
Effects of DHM on physiological and biochemical indicators of db/db mice. (**A**) Blood glucose, (**B**) TG, (**C**) TC, (**D**) LDL-C, (**E**) HDL-C, (**F**) ALT, (**G**) AST, (**H**) ROS, (**I**) GSH-Px. MET, LDHM, and HDHM vs. Control. * *p* < 0.05, ** *p* < 0.01, *** *p* < 0.001,**** *p* < 0.0001.

As shown in Figure 3A, compared with the Normal group, the blood glucose level of the Control was significantly upregulated. Compared with the Control, the blood glucose level of the MET group decreased significantly (*p* < 0.0001) and tended to be normal. The LDHM group and the HDHM group were significantly decreased (*p* < 0.001 or *p* < 0.01) in a dose-dependent manner. As shown in Figure 3B–E, the administration of DHM resulted in a significant reduction in serum levels of TC (*p* < 0.0001), TG (*p* < 0.0001), and LDL-C (*p* < 0.0001) while increasing the level of HDL-C (*p* < 0.0001) in *db*/*db* mice. These findings suggest that DHM has the potential to ameliorate blood glucose and lipid profiles, thereby addressing metabolic disturbances associated with diabetes.

The oxidative stress injury of the liver is closely related to T2DM; therefore, we investigated the DHM effects on liver oxidative stress. The results showed that DHM could significantly reduce ALT, AST, and ROS levels and significantly increase GSH-Px levels (*p* < 0.001 or *p* < 0.0001).

### 3.7. Effect of DHM on Gene Expression of Liver in db/db Mice

The Illumina NovaSeq TM 6000 sequencing platform detected a total of 112.23 GB of data. To ensure data quality control, it is required that post-filtering, Q20 should be above 90%, Q30 should exceed 85%, and the GC content should range between 40% and 50%. These criteria indicate the absence of any abnormalities in the sequencing process, ensuring the high reliability of the obtained data. After undergoing quality control screening, the sample in the 109.98 GB of valid data exhibited a minimum Q20 value of 99.98%, while the lowest Q30 value was recorded at 98.47%. Additionally, the overall QC content ranged between 47% and 48%, thereby indicating excellent sequencing data quality and high data reliability.

**Figure 4:**
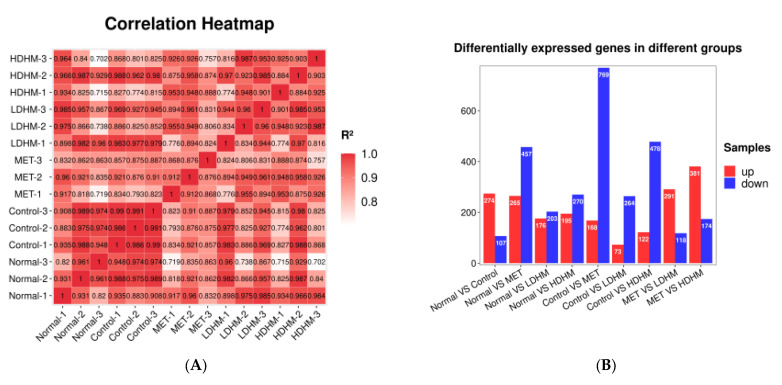

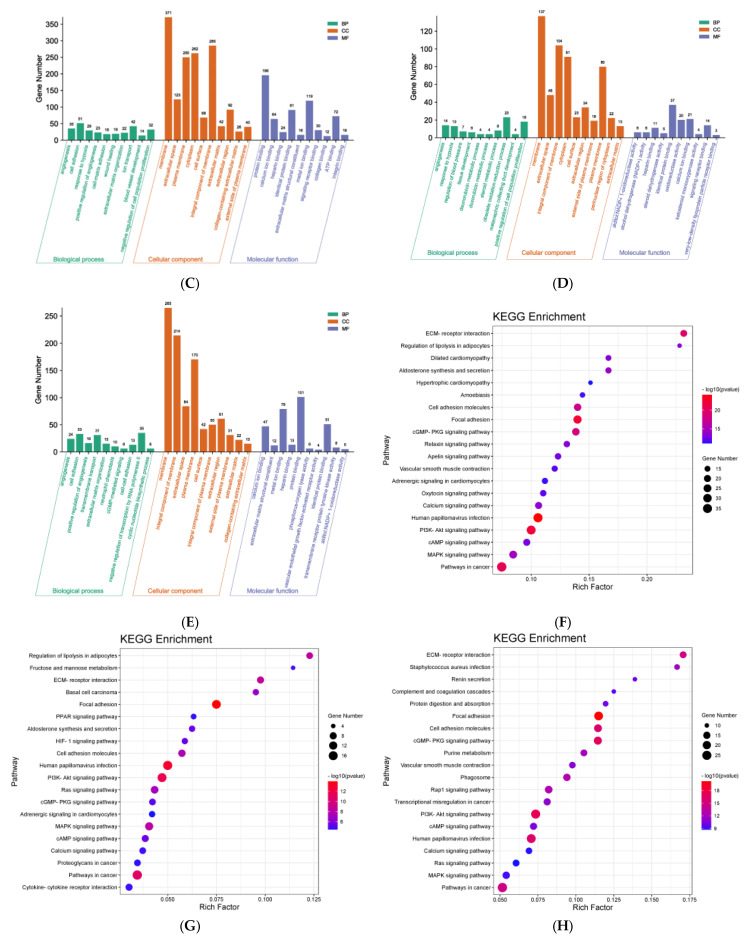
Effect of DHM on gene expression in the liver of *db*/*db* mice. (**A**) The correlation heat map between samples. (**B**) Differentially expressed genes in different groups. (**C**) GO enrichment analysis based on kidney transcriptome. MET vs. Control. (**D**) GO enrichment analysis based on kidney transcriptome. LDHM vs. Control. (**E**) GO enrichment analysis based on kidney transcriptome. HDHM vs. Control. (**F**) KEGG enrichment analysis based on kidney transcriptome. MET vs. Control. (**G**) KEGG enrichment analysis based on kidney transcriptome. LDHM vs. Control. (**H**) KEGG enrichment analysis based on kidney transcriptome. HDHM vs. Control.

The correlation among samples serves as a crucial indicator for assessing the experimental reliability and sample selection rationality. A higher METilarity in expression patterns between samples is indicated by a correlation coefficient closer to 1. We require that the R^2^ between biologically repeated samples should be at least greater than 0.8; otherwise, the sample needs to be properly explained or the experiment needs to be reperformed. The correlation heat map between samples is shown in Figure 4A. The R^2^ values of most samples in the test group were greater than 0.8, indicating that the repeatability between samples could meet the requirements of subsequent analysis.

Usually, the differentially expressed genes are screened from the aspects of fold change and significance level. Here, we use the fold change |log2FC| ≥ 1 and *p* < 0.05 as the standard to screen the differentially expressed genes. As shown in Figure 4B, there were 937 differentially expressed genes between the MET and the Control, including 168 upregulated genes and 769 downregulated genes. There were 337 differential genes between the LDHM and the Control, including 73 upregulated genes and 264 downregulated genes. In addition, there were 600 differential genes between HDHM and Control, including 122 upregulated genes and 478 downregulated genes. The number of gene expressions regulated by the HDHM group exceeds that of the LDHM group. 

### 3.8. GO Enrichment and KEGG Pathway Analysis Based on Liver Transcriptomes

As shown in Figure 4C, compared with the Control, a total of 4922 items were screened out in the MET, and a total of 1585 items were statistically significant, including 1129 items of BP, 145 items of CC, and 311 items of MF. In BP, the 10 most significant items were angiogenesis, cell adhesion, response to hypoxia, positive regulation of angiogenesis, cell-matrix adhesion, wound healing, extracellular matrix organization, ion transport, blood vessel development, and negative regulation of transcription by RNA polymerase II. In CC, the 10 most significant items were membrane, extracellular space, plasma membrane, cytoplasm, cell surface, identical protein binding, extracellular matrix, extracellular region, collagen-containing extracellular matrix, and external side of plasma membrane. In MF, the 10 most significant items were protein binding, calcium ion binding, heparin binding, identical protein binding, extracellular matrix structural constituent, metal ion binding, signaling receptor binding, collagen binding, and ATP binding.

As shown in Figure 4D, compared with the Control, a total of 2762 items were screened out in the LDHM, and a total of 1212 items were statistically significant, including 903 items of BP, 76 items of CC, and 233 items of MF. In BP, the 10 most significant items were angiogenesis, response to hypoxia, regulation of blood pressure, tissue development, daunorubicin metabolic process, doxorubicin metabolic process, steroid metabolic process, obsolete oxidation-reduction process, metanephric collecting duct development, and positive regulation of cell population proliferation. In CC, the 10 most significant items were membrane, extracellular space, an integral component of membrane, cytoplasm, cell surface, extracellular region, external side of plasma membrane, plasma membrane, perinuclear region of cytoplasm, and extracellular matrix. In MF, the 10 most significant items were alditol: NADP+ 1-oxidoreductase activity, alcohol dehydrogenase (NADP+) activity, heparin binding, steroid dehydrogenase activity, identical protein binding, oxidoreductase activity, calcium ion binding, ketosteroid monooxygenase activity, signaling receptor binding, and very low-density lipoprotein particle receptor binding. 

As shown in Figure 4E, compared with the Control, a total of 3818 items were screened out in the HDHM, and a total of 1507 items were statistically significant, including 1078 items of BP, 118 items of CC, and 311 items of MF. In BP, the 10 most significant items were angiogenesis, cell adhesion, positive regulation of angiogenesis, transmembrane transport, extracellular matrix organization, neutrophil chemotaxis, cGMP-mediated signaling, cell–cell adhesion, negative regulation of transcription by RNA polymerase II, and cyclic nucleotide biosynthetic process.

As shown in Figure 4F, the KEGG biological pathways significantly enriched in the MET were as follows: PI3K-Akt signaling pathway, cGMP-PKG signaling pathway, MAPK signaling pathway, calcium signaling pathway, cell adhesion molecules, aldosterone synthesis and secretion, regulation of lipolysis in adipocytes, regulation of lipolysis in adipocytes, vascular smooth muscle contraction, adrenergic signaling in cardiomyocyte, and cardiomyopathy. As shown in Figure 4G, the KEGG biological pathways significantly enriched in the LDHM were as follows: PI3K-Akt signaling pathway, MAPK signaling pathway, HIF-1 signaling pathway, PPAR signaling pathway, calcium signaling pathway, cell adhesion molecules, aldosterone synthesis and secretion, regulation of lipolysis in adipocytes, adrenergic signaling in cardiomyocytes, cGMP-PKG signaling pathway, and fructose and mannose metabolism. As shown in Figure 4E, the KEGG biological pathways significantly enriched in the HDHM were as follows: PI3K-Akt signaling pathway, MAPK signaling pathway, cell adhesion molecules, cGMP-PKG signaling pathway, vascular smooth muscle contraction, Ras signaling pathway, calcium signaling pathway, purine metabolism, and renin secretion. 

Through the GO and KEGG analysis results, we found that DHM could significantly affect oxidative stress, glucose metabolism, lipid metabolism-related functions, and signaling pathways, which affect these molecules’ function and the signaling pathways through treatment and intervention in the disease development of T2DM. To our surprise, we found that the following three signaling pathways were significantly enriched in MET, LDHM, and HDHM, respectively: PI3K-Akt signaling pathway, MAPK signaling pathway, and cGMP-PKG signaling pathway. 

### 3.9. qRT-PCR Analysis

**Figure 5:**
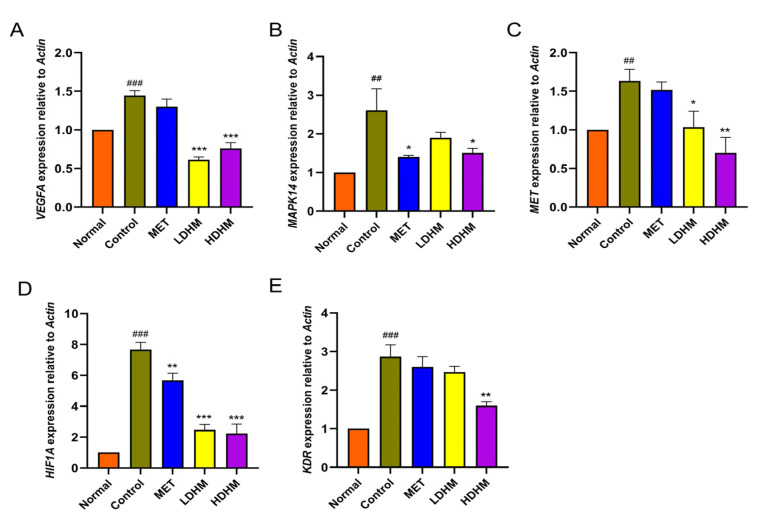
The expression of related genes and verification results. (**A**) *VEGFA*, (**B**) *MAPK14*, (**C**) *MET*, (**D**) *HIF1A*, and (**E**) *KDR*. Normal vs. Control, ^##^ *p* < 0.01; ^###^ *p*< 0.001. MET, LDHM, and HDHM vs. Control. * *p* < 0.05, ** *p* < 0.01, *** *p* < 0.001.

PI3K-Akt signaling pathway and MAPK signaling pathway were screened by network pharmacology and transcriptomics. To verify the effect of DHM on the PI3K-Akt signaling pathway and MAPK signaling pathway, qRT-PCR was used to analyze the genes related to these signaling pathways such as *VEGFA*, *MAPK14*, *MET*, *HIF1A*, and *KDR*. At the same time, it also verifies the results of molecular docking in the previous article. The results of qRT-PCR are shown in Figure 5. Compared with the Normal group, the expressions of *VEGFA*, *MAPK14*, *MET*, *HIF1A*, and *KDR* were significantly upregulated in the Control (*p* < 0.01 or *p* < 0.001). Compared with the Control, the expressions of *VEGFA*, *MET*, and *HIF1A* were significantly downregulated in the LDHM group (*p* < 0.05 or *p* < 0.001), and the expression of *MAPK14* and *KDR* showed a downward trend, but not significant. The expressions of *VEGFA*, *MAPK14*, *MET*, *HIF1A*, and *KDR* were significantly downregulated in HDHM (*p* < 0.05, *p* < 0.01 or *p* < 0.001). The HDHM could activate the PI3K-Akt signaling pathway and MAPK signaling pathway and significantly affect the expression of genes in the signaling pathway. Surprisingly, the effect of HDHM was superior to that of LDHM in a dose-dependent manner.

### 3.10. Effect of DHM on PI3K/AKT/MAPK Signaling Pathway

**Figure 6:**
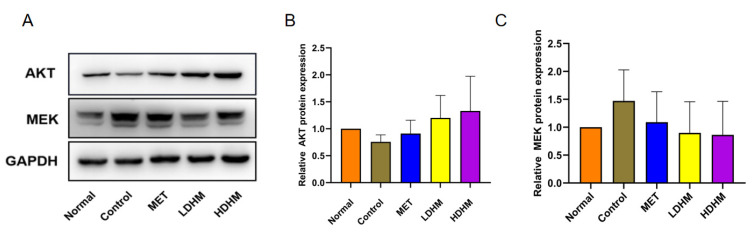
Western blotting (**A**). The relative expression of AKT (**B**) and MEK (**C**) at the protein level.

As shown in Figure 6, the Western blotting analysis revealed that DHM effectively upregulated the expression of AKT protein in liver tissue, as compared to the Control. Consequently, DHM was observed to activate the PI3K/AKT signaling pathway. Additionally, DHM demonstrated a significant downregulation of MEK expression, thereby indicating its potent inhibition of the MAPK signaling pathway. These results suggest that DHM has the potential to enhance T2DM by upregulating the PI3K/AKT signaling pathway while downregulating the MAPK signaling pathway.

## 4. Discussion

According to the definition provided by the World Health Organization (WHO), diabetes mellitus (DM) is a metabolic disorder characterized by untreated hyperglycemia resulting from inadequate insulin secretion and/or insulin resistance. The IDF Diabetes Atlas 10th edition reports that in 2021, there were approximately 537 million individuals aged 20–79 with diabetes worldwide, accounting for around 10.5% of the global population within this age group. It is projected that these numbers will increase to approximately 643 million by 2030 and reach as high as 783 million by 2045 [15]. Among all types of diabetes, type 2 diabetes mellitus (T2DM) and its complications have the highest prevalence rate, significantly impacting both healthcare systems and economies. Although hypoglycemic drugs are commonly used for treatment, they have their limitations. In recent years, natural plant extracts have gained significant attention in T2DM treatment [16]. Dihydromyricetin (DHM) is a flavonoid isolated from *Ampelopsis grossedentata*, which is traditionally used in China and exhibits various health-promoting activities such as antioxidant properties, anti-inflammatory effects, anticancer potentiality, antibacterial activity along with modulation of cell death mechanisms and regulation of lipid and glucose metabolism. Moreover, it demonstrates minimal adverse reactions [17]. DHM holds great potential as a natural product for treating and intervening in T2DM and its complications; however, its molecular mechanism regarding intervention in T2DM remains inadequately explored, which hampers its further utilization.

We employed network pharmacology and liver transcriptomics to investigate the molecular mechanisms underlying the in vitro and in vivo interventions of DHM in T2DM. Our study revealed that DHM targets several key proteins, including *VEGFA*, *MAPK14*, *MET*, *HIF1A*, *BLC*, and *KDR*. We validated these targets through molecular docking and RT-PCR. These targets primarily focus on regulating glucose metabolism, cell proliferation, apoptosis, and inflammation. *VEGFA* plays a crucial role in promoting angiogenesis and enhancing vascular permeability [18]. Numerous studies have demonstrated that serum *VEGFA* levels can effectively reflect the progression of type 2 diabetic nephropathy, with higher sensitivity and specificity observed in patients. Consequently, *VEGFA* serves as a valuable biomarker for early diagnosis and monitoring therapeutic efficacy in type 2 diabetic nephropathy [19]. *MAPK14* is a member of the mitogen-activated protein kinase (MAPK) family, which plays a crucial role in energy metabolism by inhibiting liver glycogen and fat synthesis, promoting hepatic gluconeogenesis and fatty acid oxidation, interfering with insulin signal transduction, and contributing to insulin resistance [20,21]. The *MET* receptor, widely distributed on the cell surface, engages in extracellular binding with HGF, thereby initiating the activation of multiple downstream intracellular signaling pathways, including the PI3K/AKT and RAS/RAF/MEK cascades [22]. *HIF1A* undergoes ubiquitination by ubiquitin ligase complexes and subsequent proteasomal degradation under normal conditions. However, it remains stable in hypoxia due to the requirement of molecular oxygen for its degradation mechanism. Upon activation, *HIF1A* promotes transcription of genes involved in vascular endothelial growth factor, glucose transport, and glycolysis, thereby shifting the primary energy source from mitochondrial respiration to glycolysis. The inhibition of *HIF1A* demonstrated favorable outcomes in terms of insulin content, gene expression, and the formation of mature insulin granules, thereby augmenting β-cell function [23]. 

Furthermore, DHM was found to modulate various signaling pathways such as the MAPK signaling pathway, AGE-RAGE signaling pathway implicated in diabetic complications, PI3K-Akt signaling pathway, VEGF signaling pathway, HIF-1 signaling pathway, and IL-17 signaling pathway. Our findings revealed that DHM exhibited the ability to activate this particular signaling cascade. A study has also found that DHM exerts its inhibitory effects on gluconeogenesis and glucose production by downregulating the expression of G6Pase and PEPCK via the IRS/PI3K/Akt pathway [24]. DHM represents a prototypical flavonoid compound, and numerous investigations have consistently demonstrated that the aforementioned targets and signaling pathways align with previous research on the impact of flavonoids on abnormal glucose metabolism. For instance, Scutellariae Radix and Coptidis Rhizoma exhibit down-regulatory effects on the mRNA expression of MAPK (P38, ERK, and JNK), while significantly upregulating and enhancing the phosphorylation levels of PI3K, Akt, and Glut2, thereby improving inflammation and insulin resistance in T2DM rats [25]. Didymin can activate IRS1 by increasing phosphorylation at tyrosine 895, enhance the PI3K, Akt, and GSK3, exhibit anti-diabetic complications, and promote glucose uptake through the activation of PI3K/Akt signaling pathway in insulin-resistant HepG2 cells [26]. The alkaloids, flavonoids, and polysaccharides derived from mulberry leaf exhibit hypoglycemic activity through the regulation of glucose, amino acid, and lipid metabolism. Additionally, they upregulate the expression of PPARγ, C/EBPα, and SREBP-l in 3T3-L1 cells while inhibiting agES-induced GLUTag cell damage and apoptosis via the AGEs/RAGE and p38 MAPK/NF-κB pathways [27]. The pycnogenol effectively enhances glucose uptake and contributes to the maintenance of glycemic control in 3T3-L1 adipocytes, potentially by activating the PI3K-dependent tyrosine kinase pathway involving Akt [28]. Morin exhibits inhibitory effects on the phosphorylation of p38 MAPK and JNK1/2 in mesangial cells (MCs) induced by high glucose, leading to a reduction in extracellular matrix accumulation and ultimately improving diabetic nephropathy [29].

The PI3K/AKT signaling pathway plays a crucial role in cellular mobilization, migration, differentiation, and anti-apoptotic processes. Additionally, it is involved in the regulation of glucose transport, glycogen synthesis, glycolysis, gluconeogenesis, protein synthesis, and lipolysis [30]. Furthermore, the PI3K/Akt signaling pathway has been established as a significant contributor to the pathogenesis of diabetes and its associated nephropathy [31]. The PI3K pathway is initiated by PI3K, with Akt activation playing a key role in this process through the conversion of phosphatidylinositol-4,5-diphosphate (PIP2) to phosphatidylinositol-3,4,5-triphosphate (PIP3). Akt serves as the central link in this pathway and becomes activated through phosphorylation by 3-phosphoinositide-dependent protein kinase-1 (PDK1) after translocation to the plasma membrane and undergoing structural changes using PIP3 as a substrate [32]. When insulin binds to its receptor in the body, it activates the insulin receptor tyrosine kinase, which subsequently triggers the phosphorylation of IRS-2 [33]. This phosphorylation event then leads to the activation of downstream PI3K and initiates the PI3K/Akt signal transduction pathway. The activation of Akt has been demonstrated to regulate glucose and lipid metabolism, cellular autophagy and apoptosis, inflammation, and oxidative stress by modulating the activities of glucose transporter-4 (GLUT-4), GSK-3β, forkhead box protein O1 (FoxO1), and mTOR, among others. Consequently, it plays a crucial role in the pathogenesis and progression of diabetes as well as its associated renal damage and pharmacological interventions. The Western blotting analysis demonstrated that DHM effectively upregulated the expression of AKT and activated the PI3K/AKT signaling pathway, with a more pronounced effect observed at high doses.

The MAPK kinase plays a pivotal role in promoting cell proliferation and transmitting stress signals. It is involved in the regulation of cellular processes such as proliferation, differentiation, transformation, and apoptosis through phosphorylation of nuclear transcription factors, cytoskeletal proteins, and enzymes [34]. Furthermore, it is closely associated with the development of inflammation. Recent research has demonstrated that advanced glycation end products and oxidative stress can activate the MAPK family in diabetes. Consequently, by enhancing reactive oxygen species production and inflammatory mediator release while also modulating the renin-angiotensin system and influencing glomerular mesangial extracellular matrix formation and degradation, the MAPK signaling pathway can expedite diabetic kidney disease progression [35]. Inhibitors targeting MAPK hold promise as novel therapeutic agents for diabetes and its associated kidney diseases. Surprisingly, we found that DHM was a significant MAPK inhibitor.

We must acknowledge the limitations of the present study, as the data presented may not encompass all other signaling pathways. For example, DHM was reported to mediate the AMPK-PGC-1a-SIRT3 signaling pathway to improve skeletal muscle insulin sensitivity [36] and promote autophagy by activating AMPK/mTOR signaling pathway, thereby reducing diabetic kidney injury and even delaying the progression of diabetic nephropathy [37]. DHM was found to improve glucose uptake in adipocytes by inhibiting ERK-induced phosphorylation of PPARγ at serine 273 [38]. Moreover, it was also revealed that DHM could activate the AMPK/mTOR signaling pathway, trigger autophagy, and act as the antagonism for high glucose-induced oxidative damage of endothelial cells, making it a hopeful drug for the treatment of T2DM [39]. Therefore, further investigation is warranted for these pathways. In the future, we will continue to investigate the effects of DHM targeting these signaling pathways and others on abnormal glucose metabolism and diabetes.

## 5. Conclusions

In the present study, the results of network pharmacology showed that the PI3K-Akt signaling pathway, IL17 signaling pathway, HIF signaling pathway, MAPK signaling pathway, AGE-RAGE signaling pathway in diabetic complications, and TNF signaling pathway were typical signaling pathways disturbed by DHM in T2DM. Moreover, network pharmacology also screened out the key target genes, including *VEGFA*, *SRC*, *HIF1A*, *ESR1*, *KDR*, *MMP9*, *PPARG*, and *MAPK14*. In addition, molecular docking technology was applied to verify the docking of DHM and the above targets. At the animal level, we found that DHM could significantly reduce blood glucose and improve diabetes liver oxidative stress levels. The results of transcriptomes showed that DHM could interfere with liver damage caused by T2DM by regulating redox-related enzyme activity and oxidative stress. KEGG enrichment results showed that DHM could significantly regulate the PI3K-Akt signaling pathway and MAPK signaling pathway. According to the two analyses above, we found the co-occurrence target genes in network pharmacology and transcriptomics. Therefore, RT-PCR and Western blotting were applied to verify co-occurrence target genes, and we found that DHM can significantly regulate the expressions of *VEGFA, MAPK14, MET, HIF1A*, and *KDR* in a dose-dependent manner. The Western blotting results suggest that DHM possesses the potential to augment T2DM and associated kidney injury in *db/db* mice by upregulating the PI3K/AKT signaling pathway while concurrently downregulating the MAPK signaling pathway. This study provides a basis for DHM as a new drug for type 2 diabetes. In addition, it also provides a new strategy for the prevention and treatment of T2DM.

## Figures and Tables

**Table 1:** Genes associated with signaling pathways.

KEGG ID	Pathway	Genes	*p*-Value	Fold Enrichment
hsa01522	Endocrine resistance	SRC, MMP2, BCL2, MAPK14, ESR1, MMP9, ESR2	1.21 × 10^−06^	18.79262673
hsa05418	Fluid shear stress and atherosclerosis	SRC, MMP2, BCL2, KDR, MAPK14, MMP9, VEGFA	9.33 × 10^−06^	13.24947784
hsa04014	Ras signaling pathway	PLA2G1B, KIT, PLA2G2A, KDR, MET, FGFR1, VEGFA	1.78 × 10^−04^	7.836925189
hsa04010	MAPK signaling pathway	KIT, KDR, MAPT, MAPK14, MET, FGFR1, VEGFA	5.96 × 10^−04^	6.264208909
hsa04933	AGE-RAGE signaling pathway in diabetic complications	STAT1, MMP2, SERPINE1, BCL2, MAPK14, VEGFA	2.80 × 10^−05^	15.78580645
hsa04151	PI3K-Akt signaling pathway	KIT, BCL2, KDR, MET, FGFR1, VEGFA	0.008711778	4.459267359
hsa05417	Lipid and atherosclerosis	SRC, BCL2, PPARG, MAPK14, MMP9	0.007515789	6.118529632
hsa05208	Chemical carcinogenesis—reactive oxygen species	SRC, MAPK14, HIF1A, MET, VEGFA	0.008531633	5.899030812
hsa04370	VEGF signaling pathway	SRC, KDR, MAPK14, VEGFA	0.001270708	17.83706944
hsa04066	HIF-1 signaling pathway	SERPINE1, BCL2, HIF1A, VEGFA	0.007251828	9.654927493
hsa05415	Diabetic cardiomyopathy	MMP2, MAPK14, PPARA, MMP9	0.037668717	5.184172891
hsa00590	Arachidonic acid metabolism	PLA2G1B, PLA2G2A, PTGS1	0.020925862	12.93918562
hsa04657	IL-17 signaling pathway	MMP13, MAPK14, MMP9	0.046394807	8.396705559
hsa00564	Glycerophospholipid metabolism	ACHE, PLA2G1B, PLA2G2A	0.049997898	8.053982883
hsa04668	TNF signaling pathway	MMP14, MAPK14, MMP9	0.063365979	7.047235023

**Table 2:** Molecular docking results of DHM and key targets.

Composition	Targets	PBDID	Affinity (kcal/mol)
DHM	VEGFA	1VPF	−7.0
	SRC	4M4Z	−6.4
	MAPK14	5ETI	−9.0
	MMP9	5TH9	−10.2
	MET	6I04	−7.9
	HIF1A	1h2k	−7.5
	KDR	1ywn	−7.5
	PPARG	2VV2	−8.2

## Data Availability

Data is contained within the article or Supplementary Materials.

## Supplementary Materials

The following supporting information can be downloaded at: https://www.mdpi.com/article/10.3390/foods13020344/s1, Figure S1: Quantification of DHM content by using HPLC, Table S1: List of primer sequences, Table S2: The topology analysis of PPI network.
