# Supplementary material for: The Molecular Mechanism Underlying the Therapeutic Effect of Dihydromyricetin on Type 2 Diabetes Mellitus Based on Network Pharmacology, Molecular Docking, and Transcriptomics
Source: Foods. 2024 Jan 22;13(2):344. doi: 10.3390/foods13020344 (PMC10815645; doi:10.3390/foods13020344)
Supplement: Supplementary file 1 [file foods-13-00344-s001.zip › foods-2808754-supplementary.pdf]

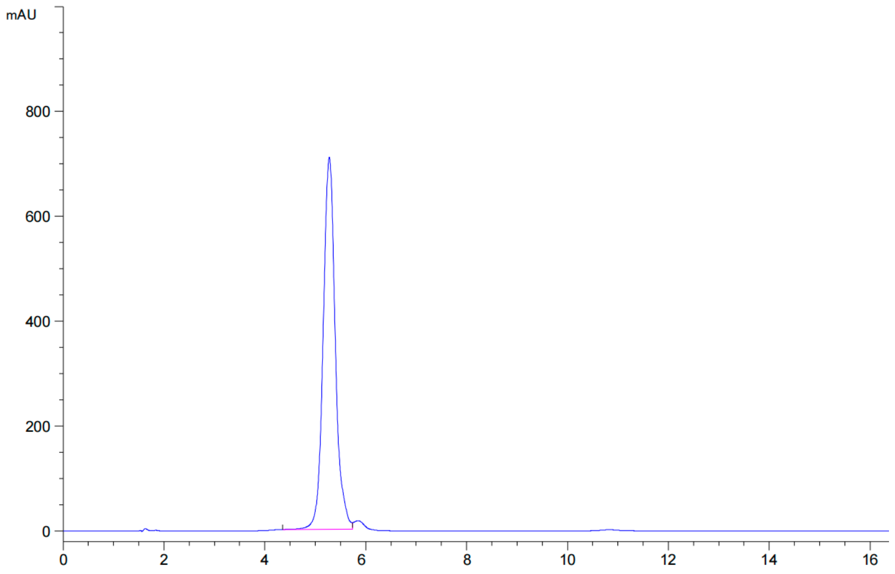

Figure S1. Quantification of DHM content by using HPLC.

Table S1. List of primer sequences.

Gene Name	Primer Sequences（5′—3′）
VEGFA	F: TTGCCTTGCTGCTCTACCTCCA<br>R: GATGGCAGTAGCTGCGCTGATA
MAPK14	F:GAGCGTTACCAGAACCTGTCTC<br>R:AGTAACCGCAGTTCTCTGTAGGT
HIF1A	F:TATGAGCCAGAAGAACTTTTAGGC<br>R: CACCTCTTTTGGCAAGCATCCTG
KDR	F:GGAACCTCACTATCCGCAGAGT<br>R:CCAAGTTCGTCTTTTCCTGGGC
MET	F:TGCACAGTTGGTCCTGCCATGA<br>R:CAGCCATAGGACCGTATTTCGG

Table S2. The topology analysis of PPI network.

	**Gene name**	**Betweenness**	**Closeness**	**Degree**
1	VEGFA	128.45741	0.8	48
2	SRC	138.58505	0.7619048	46
3	HIF1A	88.57314	0.7619048	46
4	ESR1	93.6636	0.72727275	42
5	KDR	20.187223	0.6666667	34
6	MMP9	37.36483	0.6666667	34
7	PPARG	80.0737	0.6666667	32
8	MAPK14	77.58244	0.6530612	30
9	KIT	5.0702105	0.58181816	24
10	MET	5.1237907	0.58181816	24
11	APP	61.06575	0.6037736	24
12	MMP2	4.7783885	0.6037736	24
13	SERPINE1	17.940353	0.5925926	24
14	STAT1	2.1776557	0.5714286	20
15	ABCG2	8.20901	0.55172414	18
16	MMP14	3.4539683	0.55172414	18
17	ESR2	10.915079	0.5614035	18
18	TERT	3.8014653	0.55172414	18
19	FGFR1	0.78571427	0.5423729	18
20	ABCB1	6.676083	0.5423729	16

21	PPARA	2.4277778	0.55172414	16
22	CYP19A1	6.825275	0.53333336	14
23	MMP13	1.4611111	0.53333336	14
24	DNMT1	0.83956045	0.50793654	12
25	CES1	1.5660173	0.45070422	8
26	MAPT	8.884127	0.4923077	8
27	SHBG	0.6666667	0.47058824	8
28	PLA2G1B	25.571428	0.45714286	8
29	PTGS1	37.27316	0.4848485	8
30	ACHE	0	0.38554215	4
31	BCL2	0	0.45714286	4
32	PLA2G2A	0	0.34408602	4
33	KCNH2	0	0.43835616	2
